# Screening Anti-Inflammatory Effects of Flavanones Solutions

**DOI:** 10.3390/ijms22168878

**Published:** 2021-08-18

**Authors:** Paola Bustos-Salgado, Berenice Andrade-Carrera, Valeri Domínguez-Villegas, Natalia Díaz-Garrido, María J. Rodríguez-Lagunas, Josefa Badía, Laura Baldomà, Mireia Mallandrich, Ana Calpena-Campmany, María Luisa Garduño-Ramírez

**Affiliations:** 1 Department of Pharmacy and Pharmaceutical Technology and Physical Chemistry, Faculty of Pharmacy and Food Science, University of Barcelona, Av. Joan XXIII 29-31, 08028 Barcelona, Spain; pbustosa19@alumnes.ub.edu (P.B.-S.); mireia.mallandrich@ub.edu (M.M.); 2 Faculty of Chemical Sciences and Engineering, Autonomous University of the State of Morelos, Av. Universidad 1001, Cuernavaca 62209, Mexico; bereniceac@uaem.mx (B.A.-C.); valeri.dominguez@uaem.mx (V.D.-V.); 3 Department of Biochemistry and Physiology, Faculty of Pharmacy and Food Sciences, University of Barcelona, 08028 Barcelona, Spain; natalia.diaz.garrido@gmail.com (N.D.-G.); mjrodriguez@ub.edu (M.J.R.-L.); josefabadia@ub.edu (J.B.); lbaldoma@ub.edu (L.B.); 4 Institute of Biomedicine, University of Barcelona (IBUB), 08028 Barcelona, Spain; 5 Research Institute Sant Joan De Déu (IR-SJD), University of Barcelona (IBUB), 08028 Barcelona, Spain; 6 Institute of Research in Food Nutrition and Safety, University of Barcelona (INSA-UB), 08921 Santa Coloma de Gramenet, Spain; 7 Center for Chemical Research, Institute for Research Basic and Applied Sciences, Autonomous University of the State of Morelos, Av. Universidad 1001, Cuernavaca 62209, Mexico

**Keywords:** flavanones, *Eysenhardtia platycarpa*, in vivo anti-inflammatory activity, cytokines

## Abstract

There are a large number of remedies in traditional medicine focused on relieving pain and inflammation. Flavanones have been a potential source in the search for leading compounds and biologically active components, and they have been the focus of much research and development in recent years. *Eysenhardtia platycarpa* is used in traditional medicine for the treatment of kidney diseases, bladder infections, and diabetes mellitus. Many compounds have been isolated from this plant, such as flavones, flavanones, phenolic compounds, triterpenoid acids, chalcones, sugars, and fatty acids, among others. In this paper, natural flavanone **1** (extracted from *Eysenhardtia platycarpa*) as lead compound and flavanones **1a**–**1d** as its structural analogues were screened for anti-inflammatory activity using *Molinspiration*^®^ and *PASS Online* in a computational study. The hydro alcoholic solutions (FS) of flavanones **1**, **1a**–**1d** (FS**1**, FS**1a**–FS**1d**) were also assayed to investigate their in vivo anti-inflammatory cutaneous effect using two experimental models, a rat ear edema induced by arachidonic acid (AA) and a mouse ear edema induced by 12-*O*-tetradecanoylphorbol acetate (TPA). Histological studies and analysis of pro-inflammatory cytokines TNF-α, IL-1β, and IL-6 were also assessed in AA-inflamed rat ear tissue. The results showed that the flavanone hydro alcoholic solutions (FS) caused edema inhibition in both evaluated models. This study suggests that the evaluated flavanones will be effective when used in the future in skin pathologies with inflammation, with the results showing **1b** and **1d** to be the best.

## 1. Introduction

Inflammation is a nonspecific immune response against injury by harmful agents that attempts to restore homeostasis and the function of damaged tissues. The first reaction to external agents is an acute inflammation produced by accumulation of leukocytes that migrate from the blood to the damaged sites. Recruited immune cells release high levels of cytokines and other mediators. Upregulation of these inflammatory mediators mainly depends on cell signaling transduction cascades that lead to activation of NF-κβ, a central transcription factor that controls the expression of cytokines, chemokines, and adhesion molecules relevant for the inflammatory process [1,2].

For treatment of skin conditions, topical application is an effective drug administration route that allows the delivery of high drug concentrations to the damaged sites. Specific therapies to treat inflammation with non-steroidal drugs (NSAIDs), or glucocorticoids, show a variety of side effects with long-term use, making these drugs unsuitable for chronic therapies [3,4,5]. These effects include cutaneous atrophy and the rebound of the disease on discontinuation of use, among other effects [6]. Plant-derived natural compounds may potentially be suitable in this regard [4,7]. Since ancient times, many people who suffer from a variety of inflammatory conditions have been treated with products derived from plants. Natural products offer therapeutic alternatives because of their ability to produce various bioactive metabolites that are difficult to synthesize. In fact, it is estimated that 40% of approved drugs originate from or have been inspired by natural products [8,9,10,11].

It has been shown that some natural flavonoids present anti-inflammatory activity in vivo after oral or dermal administration [2]. Increasing scientific evidence has shown that flavonoids can have anti-inflammatory properties by inhibiting regulatory enzymes, among others, necessary for controlling mediators involved in inflammation such as phospholipase A_2_, COX, and lipoxygenase (LOX) [2]. It was recently discovered that certain flavonoid derivatives modulate the expression level of several genes associated with inflammation. In particular, flavonoids modulate multiple kinases of immune signaling pathways and inhibit NF-κβ, thus reducing cytokine expression [12,13]. In addition, flavonoids inhibit maturation of dendritic cells, and consequently reduce cytokine secretion and the proliferation of T cells. Due to their phenolic structure, flavonoids are good free radical scavengers. Because reactive oxygen species (ROS) are produced at high levels in inflamed tissues, the antioxidant activity also contributes to the anti-inflammatory potential of flavonoids [2]. In addition, some derivatives of flavonoids inhibit the production of pro-inflammatory cytokines such as TNF-α [14].

Flavanones, a group of polyphenolic compounds, have been the focus of intense research and development due to their wide range of biological activities, including anti-inflammatory effects [15,16]. Mexico has a rich tradition in using ethno-medicine. In recent decades, the *Eysenhardtia* genus has attracted attention due to its medicinal properties. From this family of plants, many compounds have been isolated, such as flavones, isoflavones, flavanones, phenolic compounds, chalcones, dihydrochalcones, coumarins, pterocarpan, sugars, and fatty acids, among others. Several pharmacological studies have been linked to the identification of its health benefits, highlighting diuretic, antidiabetic, antiglycation, antioxidant, anti-inflammatory, antimicrobial potential, and cytotoxic properties [17]. Narvaez-Mastache et al. demonstrated the antioxidant and anti-hyperglycemic activities of 3-*O*-Acetyl-11α,12α-epoxy-oleanan-28,13β-olide isolated from *Eysenhardtia platycarpa*, *E. punctata*, and *E. subcoriacea* (Fabaceae) species [18]. In another report, Wächter et al. isolated two prenylflavanones (4′,5,7-trihydroxy-8-methyl-6-(3-methyl-[2-butenyl])-(2*S*)-flavanone and 4′,5,7-trihydroxy-6-methyl-8-(3-methyl-[2-butenyl])-(2*S*)-flavanone) from *Eysenhardtia texana* Kunth and showed the growth inhibition of *Staphylococcus aureus* and *Candida albicans* in an agar-gel diffusion assay [19]. Furthermore, Pérez-Gutierrez et al. found that isoflavones (7-hydroxy-2′,4′,5′-trimethoxyisoflavone and 7-hydroxy-4′-etoxyisoflavone) isolated from the bark of *Eysenhardtia polystachya* were able to inhibit the formation of calcium phosphate crystals, and consequently reduced the appearance of kidney stones, thus suggesting the use of isoflavones as a preventive treatment [20]. Additionally, the isoflavanes, (3S)-7-hydroxy-2′,3′,4′,5′,8-pentamethoxyisoflavan and (3S)- 3′,7-dihydroxy-2′,4′,5′,8-tetramethoxyisoflavan, isolated from *Eysenhardtia polystachya,* showed slight cytotoxic activity against the cell lines KB (nasopharyngeal carcinoma), P388 (murine leukemia), and SQC-1 UISO (squamous cell carcinoma of the cervix) [21]. Recently, an in vivo study has evidenced that five flavanones (5,7-dihydroxy-6-methyl-8-prenylflavanone, 5,7-dihydroxy-8-methyl-6-prenylflavanone, 5,7-dihydroxy-6-prenylflavanone, 5,7-dihydroxy-8-prenylflavanone, and 5-hydroxy-7-methoxy-8-prenylflavanone) isolated from a methanolic extract of *Eysenhardtia platycarpa* showed an anti-inflammatory effect [22].

The immense structural diversity of natural compounds and their potential bioactivity allow the application of pharmacomodulation methods to improve their therapeutic potential. Pharmacomodulation consists of taking a chemical substance with a known structure and recognized biological activity to design and test structural analogues with greater pharmacological activity and fewer side effects. Flavanones have been a potential source in the search for lead compounds and biologically active components [23]. This process can be complemented with computational chemistry to offer compounds that are far more efficient than those currently used in clinical practice [24,25].

**Figure 1:**
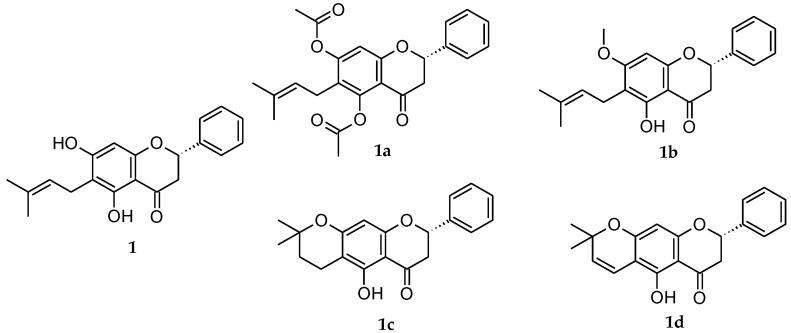
Natural extracted flavanone **1** and its structural analogues flavanones **1a**–**d**.

Against this background, we investigated the in vivo anti-inflammatory effects of four flavanone derivatives **1a**–**1d** (Figure 1) in solution (FS**1**, FS**1a**–FS**1d**) obtained from the derivatization process by molecular modification of flavanone **1** extracted from *E*. *platycarpa* to generate structural analogues with greater pharmacological activity. *Molinspiration*^®^ and *PASS* (Prediction of Activity Spectra for substance) *Online* analyses were performed for the derivatization compounds in order to find out whether they possess the drug-like character or not before in vivo analysis. The therapeutic efficacy of flavanones was evaluated in the TPA (12-*O*-tetradecanoylphorbol-13-acetate) edema mouse and arachidonic acid (AA) edema rat models. In addition, histological analysis and quantification of cytokine mRNA expression (TNF-α, IL-1β, and IL-6) was evaluated in AA-inflamed rat ears.

## 2. Results

### 2.1. In Silico Analyses

The freely accessible web resource *PASS Online* was used to evaluate the flavanones **1**, **1a**–**d** before in vivo assays. The output information of flavanones‘ potential activity as anti-inflammatory agents “*Pa*” is given in Table 1.

Additionally, the activity of the natural **1** and derivatives flavanones **1a**–**d** was analyzed under *Molinspiration*^®^ bioactivity criteria. Results are shown also in Table 1 by means of numerical assignment.

### 2.2. Model of Mice Ear Inflammation Induced with TPA

The HPLC (high-performance liquid chromatography) results showed great short-term stability. Above 95% of the original amounts of flavanones remained in the solution after 5 days storage. This short-term stability ensured that the quantity of flavanones had not been altered before their use in the in vivo experiments. Table 2 displays the results of the in vivo anti-inflammatory evaluation of the flavanones as mean values ± standard deviation (SD). The flavanone natural **1** yielded a significant reduction of the dermal edema, with an inhibition percentage of 66.67 ± 1.55. However, only the flavanone derivative **1d** showed an inhibition percentage (96.27 ± 1.93) higher than the indomethacin value (91.35 ± 0.47).

### 2.3. Model of Rat Ear Inflammation Induced with AA and Anti-Inflammatory Response after Flavanone Solution Treatment

**Figure 2:**
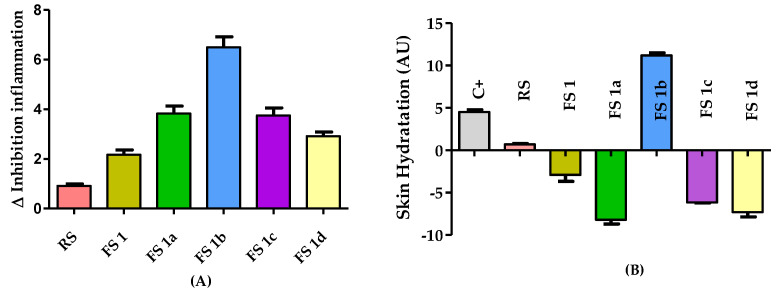
(**A**) Anti-inflammatory activity evaluated after FS treatment in AA-induced edema model showed in the increment or decrement of thickness with respect to initial conditions. (**B**) Skin hydration results after the application of FS treatments in AA-induced rat ear edema are shown as the difference in hydration compared to initial conditions. Results are expressed as mean ± SD (*n* = 5). C+ = positive control, RS= reference drug solution, FS = flavanone solution (**1**, **1a**–**1d**).

The edema reduction, associated with the flavanone solutions FS**1**, FS**1a**–FS**1d** treatment in an in vivo rat ear model of inflammation induced by AA, was evaluated by the difference in thickness measured before and after the topical application of the flavanone solutions. A solution of the reference drug (diclofenac sodic) was also evaluated (RS). The results are depicted in Figure 2A. The FS reduced the ear thickness more than the RS. Moreover, the FS**1a** with the FS**1c** achieved almost the same edema reduction. Of note, the FS**1b** displayed the highest efficacy because it reduced the thickness of the rat ears after 20 min of application.

Figure 2B shows the skin hydration results for rat ears as the difference in stratum corneum hydration (SCH) after the FS treatments and the basal conditions as arbitrary units (AU). All the treatments reduced the skin hydration, but FS**1b** was an effective treatment and able to increase skin hydration at the same time.

**Figure 3:**
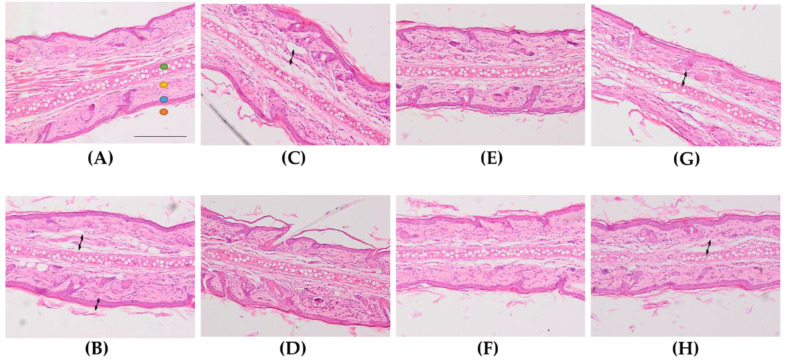
Representative micrographs of rat’s ear (x100 magnification). (**A**): Control −, (**B**): Control +, (**C**): reference drug solution (RS), (**D**): FS**1**, (**E**): FS**1a**, (**F**): FS**1b**, (**G**): FS**1c**, (**H**): FS**1d**. 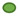 auricular cartilage, 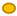 dermis, 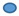 epidermis, 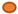 stratum corneum. Arrows indicate presence of edema. Scale bar = 200 µm.

For the assessment of the anti-inflammatory activity of the formulations, histological analysis of ear sections was performed (Figure 3). A mild inflammation was observed in ears treated with AA (Figure 3B), characterized by edema and increased epidermal thickness. Topical administration of the reference drug slightly decreased these inflammatory indicators. Histological analysis of the ear of FS-treated animals showed different degrees of reduction of the edema (Figure 3D–H). In this regard, FS**1b** (Figure 3F) was the best formulation in diminishing the inflammation induced by AA, showing even better results than the reference drug (Figure 3C). Moreover, FS**1** and FS**1a** also showed less edema, although FS**1** showed greater presence of leukocyte infiltrate. Furthermore, FS**1c** and FS**1d** were also able to reduce the edema but to a lower degree than FS**1** and FS**1a**.

**Figure 4:**
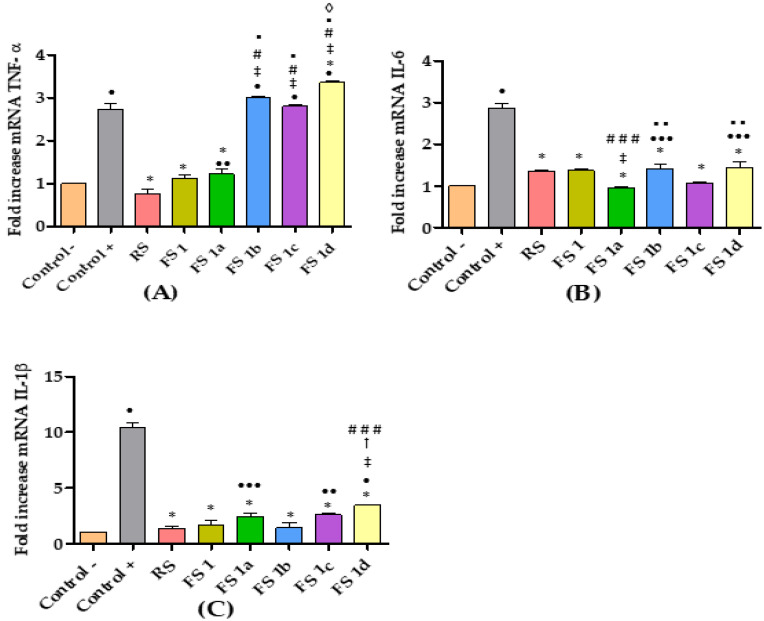
Relative expression of cytokines: (**A**) TNF-α; (**B**) IL-6; (**C**) IL-1β. Non-treated rats (Control −), rats treated only with AA (Control +), rats treated with diclofenac sodic solution (reference anti-inflammatory drug) after inducing inflammation (RS), rats treated with flavanone solutions FS**1**, FS**1a**- FS**1d**. Significant difference between Control + (*), Control − (•), RS (‡), FS**1** (#), FS**1a** (▪), FS**1b** (†), FS**1c (◊);** (**###,**•••) *p* < 0.05; (▪ ▪, ••) *p* < 0.01; (*, •, ‡, #, ▪, †, ◊) *p* < 0.001 by non-parametric Tukey’s *t*-test (*n* = 5).

For better understanding of the topical anti-inflammatory effect, FS**1** and FS**1a**–**1d** were tested for the cytokine reduction in edema-inflamed ear tissue. Results from the RT-qPCR analysis are presented in Figure 4. Rat ear samples from the positive control group showed significantly higher mRNA expression of pro-inflammatory cytokines IL-6, IL-1β, and TNF-α than samples from non-treated rats (negative control), thus confirming the inflammatory effect of AA. A trend of reduced expression of the pro-inflammatory cytokines IL-6 and IL-1β was also observed with the application of all the flavanone solutions as well as the reference solution (RS) (Figure 4B,C). Regarding TNF-α, only the FS**1** and FS**1a** diminished the expression of this cytokine to levels that were not significantly different from those of the negative control group. Moreover, no significant differences were observed between FS**1** and FS**1a** results (Figure 4A).

## 3. Discussion

The search for more effective therapeutic agents with less undesirable side effects to treat and reduce the signs and symptoms of acute and chronic inflammatory diseases is still a challenge. Although the use of synthetic anti-inflammatory agents is often very effective, there is great interest in plant-based anti-inflammatory medicine.

The PASS computer program allows estimating the probable profile of the biological activity of a drug-like organic compound based on its structural formula [26,27]. The prediction of the biological activity spectra obtained using the *PASS Online* computer program showed that all the flavanones were predicted to display anti-inflammatory action and have a probability of being active (*Pa*) of up to 0.6, similar to the common drug references (Table 1). The in vivo anti-inflammatory evaluation was carried out with the aim of confirming these predictions. Molecular properties such as lipophilicity, molecule size, flexibility, and so on, influence the behavior of compounds in a living organism, including their bioavailability, transport properties, affinity to proteins, reactivity, and many others [28]. To obtain more information on the anti-inflammatory therapeutic profile of flavanones, some virtual studies were carried out and compared with the values obtained with standard drugs. Therefore, the prediction of Log*P*, molecular polar surface area (PSA), and other physicochemical properties were calculated using *Molinspiration*^®^ software programs to predict the flavanones’ (**1**, **1a**–**1b**) bioactivity. The most representative results are summarized in Table 1. The Log*P* (octanol/water partition coefficient) is used in drug design as a measure of molecular lipophilicity. Lipophilicity affects drug absorption, bioavailability, hydrophilic drug-receptor interactions, and metabolism of molecules, as well as their toxicity. The method for predicting Log*P* developed in *Molinspiration***^®^** (miLog*P*) is one of the best methods available for predicting this parameter. It has been reported that there is a very good correlation between *Molinspiration*^®^ calculated log*P* and several drug transport properties [29]. The calculated miLog*P* values for the investigated flavanones (**1**, **1a**–**1b**) were in the range of 3.82 to 4.57 (Table 1), which were acceptable values. Thus, these compounds were expected to present good bioavailability. Molecular polar surface area (TPSA) is a very useful descriptor for predicting drug absorption and transport properties, for which the area of the polar surface is defined as the sum of the surfaces of the polar atoms such as oxygen, nitrogen, and hydrogen attached to a molecule. Therefore, TPSA is a molecular property related to the polarity, hydrogen-bonding potential, and water solubility of organic molecules. Molecules with TPSA values around 160 or more are expected to exhibit problems in crossing the membrane barriers [30]. In this regard, the TPSA values of flavanones were less than 78.92 (Table 1). Therefore, this meant they could efficiently cross the skin.

Repeated and prolonged inflammation is related to a wide range of progressive diseases, including inflammatory conditions, autoimmune disorders, and cancers [31]. The design of new and safe molecules able to reduce the inflammation could be of great interest. To determine the structural requirements for the flavanones to exert anti-inflammatory activity, it is necessary to obtain a varied series of these compounds and to test them. The induction of edema in rodent’s ears is an in vivo model of acute inflammation that has been widely used in the investigation of topical anti-inflammatory activity [32]. Inflammation is induced by irritants, of which AA and TPA are the most widely used [33]. The results obtained from in vivo assays with TPA and AA edema animal models (Table 2 and Figure 2) make clear that the natural flavanone solution FS**1** and the structural analogue flavanone solutions (FS**1a**–FS**1d**) have topical anti-inflammatory activity, and of these flavanones, FS**1b** and FS**1d** stand out. The five FS were capable of reducing the epidermal thickness in the AA-treated ear model. The better anti-inflammatory effect displayed by FS**1b** and FS**1d** suggested that the molecular modification of flavanone **1** improved the anti-inflammatory activity. As in previous studies [34], these results again confirm that a planar ring system and hydroxyl groups at the 5- and 7-position of the A-ring seem to be vital in the flavonoid molecules, which means that they exhibit the anti-inflammatory action revealed in SARs studies [35,36]. These facts could explain why the structure of flavanone **1b,** which possesses a hydroxyl group at the 5- position, and flavanone **1d,** with a more rigid structure, were favored for the anti-inflammatory effect in the models evaluated in this work. In the future, these results will provide enough data to obtain a reliable structure-activity relationship.

In effect, a marked difference exists in the mechanisms of the inflammatory response induced by topical application of TPA and AA in the rodent. It is well established that when TPA was applied to the animal ear, it produced low and long-lasting edema with pronounced tissue damage due to the participation of multiple pathways. Moreover, the vascular permeability paralleled the influx of neutrophils. Conversely, the AA- induced effect was rapid but of short duration, and the vascular permeability preceded the neutrophil influx. Furthermore, TPA is associated with protein kinase C (PKC) stimulation, which in turn initiates other enzymatic cascades such as mitogen-activated protein kinases (MAPK) and phospholipase A_2_ (PLA_2_) that trigger the release of free AA and lead to the production of prostaglandins (PGE_2_) and leukotrienes (LT), among others. Whereas AA is associated with marked increases in the levels of oxidized fatty acids and the release of inflammatory mediators such as histamine, prostaglandins, and leukotrienes, phospholipase A_2_ and cyclooxygenase (COX) inhibitors have little or no effect [37]. These models can be used to identify compounds that interfere with AA metabolism and thus investigate the mechanisms of lipoxygenase (LOX) inhibition [38]. However, several studies have shown that AA-induced inflammation can be inhibited by mechanisms other than COX/LOX enzyme inhibition, such as the modulation of histamine and serotonin response [32,33,37,39,40,41,42,43,44]. At this stage of our work, it is difficult to speculate about the exact mechanism through which flavanones **1**, **1a**–**1d** exert such an effect.

Cytokines are regulatory proteins that play a key role in inflammatory responses [45,46]. The inhibition of some pro-inflammatory cytokines can alleviate many inflammatory diseases [47,48,49,50]. It is reported that flavonoids inhibit pro-inflammatory mediators such as cytokines/chemokines, eicosanoids, and adhesion molecules [51,52]. In this sense hesperidin, a flavanone isolated from citrus fruit, has anti-inflammatory and antioxidant properties. The hesperidin anti-inflammatory effects are correlated with inhibition of pro-inflammatory mediators, including cytokines (IL-8, IL-6, TNF-α, IL-1β), oxidative stress enzymes (iNOS, COX-2), prostaglandin, and intercellular adhesion molecules (ICAM) [53,54]. Continuing with our interest in the evaluation of the biological potential of flavanones [55], and in order to prove their anti-inflammatory effects, the gene expressions of IL-1β, IL-6, and TNF-α were analyzed in small sections of the rat ear tissue after FS**1**, FS**1a**- FS**1d** treatment. Overall, in our investigation, the FS**1**, FS**1a**–FS**1d** inhibited the production of IL-1β and IL-6 with respect to the positive control (Figure 4B,C). In addition, the FS**1b** showed better results when IL-1β was evaluated. Interestingly, FS**1** and FS**1a** were more effective than other assayed flavanones in inhibiting the AA-induced production of TNF-α, while all of them were effective in inhibiting the AA-induced production of IL-6 and IL-1β. Again, these results suggest that flavanones can target different signaling pathways in a structure-dependent manner [56]. Our results suggest that flavanones are efficient in inflammatory skin disorders.

## 4. Materials and Methods

### 4.1. Materials

Propylene glycol monolaurate-type II (Lauroglycol™ 90), ethanol analytical grade, and reagents for histological procedures were purchased from Sigma-Aldrich (Madrid, Spain) and Thermo Fisher Scientific (Barcelona, Spain). Acetonitrile (AcN) HPLC grade (Fisher Scientific, Barcelona, Spain) was filtered with a 0.45 µm PVDF membrane filter (Millipore Corp., Madrid, Spain). A Millipore Milli-Q^®^ purification system (Millipore Corporation; Burlington, Middlesex, MA, USA) was used to obtain purified water for all experiments. Polyglyceryl-6-dioleate (Plurol^®^ oleique), triglyceride medium-chain EP/NF/JPE (Labrafac^®^ lipophile), and caprylocaproyl macrogol-polyoxyl-8-glyceride (Labrasol^®^) were supplied by Gattefossé (Saint-Priest, Lyon, France).

### 4.2. Plant Extraction and Derivatization of Flavanones

The extraction and isolation of flavanone **1** ((2*S*)-5,7-dihydroxy-6-(3-methyl-2-buten-1-yl)-2-phenyl-2,3-dihydro-4*H*-1-Benzopyran-4-one) shown in Figure 1, were described previously [34]. Briefly, *E. platycarpa* leaves were collected from the municipality of Tetipac, Guerrero State (Mexico), and were then kept in the Faculty of Herbarium, Facultad de Ciencias de la Universidad Nacional Autónoma de México. Professor Ramiro Cruz (voucher specimen 1325) authenticated the plant material. For every 100 g of dried leaves of *E. platycarpa*, 1000 mL of methanol (Sigma-Aldrich, Toluca de Lerdo, Mexico) were added to obtain the corresponding methanolic extract, and then they were concentrated *in vacuo* to obtain the crude extracts. Finally, the flavanone **1** was isolated by column chromatography at reduced pressure and purified by using thin-layer chromatography (TLC). The obtained yellow powder precipitate was characterized by comparison with previously published melting point data and with ^1^H-NMR results [57]. The flavanones **1a**–**1d** were obtained in accordance with the method previously reported [57] through esterification, methylation, cyclization, and vinylogous-cyclization reactions. The resulting derivative flavanones were ester derivative **1a** ((2*S*)-5,7-bis(acetyloxy)-6-(3-methyl-2-buten-1-yl)-2-phenyl-2,3-dihydro-4*H*-1-Benzopyran-4-one); ether derivative **1b** ((2*S*)-5-hydroxy-7-methoxy-6-(3-methyl-2-buten-1-yl)-2-phenyl-2,3-dihydro-4*H*-1-Benzopyran-4-one); cyclized derivative **1c** ((8*S*)-5-hydroxy-2,2-dimethyl-8-phenyl-3,4,7,8-tetrahydro-2*H*,6*H*-Benzo[1,2-*b*:5,4-*b*′]dipyran-6-one); and vinylogous cyclized derivative **1d** ((8*S*)-5-hydroxy-2,2-dimethyl-8-phenyl-7,8-dihydro-2*H*,6*H*-Benzo[1,2-*b*:5,4-*b*′]dipyran-6-one) (Figure 1).

### 4.3. In Silico Analysis

The computational methods (in silico) are some of the well-known approaches that have been used regularly to produce the 3D models to identify physicochemical properties and to predict biological activities [58]. Molinspiration^®^ server (http://www.molinspiration.com accessed on 18 February 2021) and PASS Online (Prediction of Activity Spectra for substance, http://way2drug.com/PassOnline accessed on 18 February 2021) were used to predict the bioactivity [26,59] of the flavanones **1**, **1a**–**d**.

### 4.4. Flavanone Solutions (FS)

The flavanone solutions (FS**1**, FS**1a**–FS**1d**) were prepared by dissolving 5.0 mg of the respective flavanone (**1**, **1a**, **1b**, **1c** and **1d**) with 1 mL of the mixture of EtOH/H_2_O (7:3). The purity and chemical stability of dissolutions were examined after 5 days of their preparation storage at 4 ± 1 °C using the validated analytic method described before in terms of linearity, accuracy, and precision [60]. Briefly, the flavanones were determined by high performance liquid chromatography (HPLC) on a Waters system, equipped with a Waters 515 HPLC pump, a 717 Plus autosampler, and a dual λ absorbance UV-vis 2487 detector (Waters, Milford, Worcester, MA, USA). The chromatographic column used was a reversed phase column Atlantis^®^ C18 (5 µm 250 mm × 4.6 mm, Waters). The separations were carried out with a flow rate of 1 mL/min under isocratic elution. The mobile phase was W-water and AcN-acetonitrile (% W: % AcN) with a different composition for each flavanone: **1** (30:70), **1a** (20:80), **1b** (40:60), **1c** (20:80) and **1d** (10:90) and detection wavelength at 300 nm for **1**, **1b**, **1c**, **1d**, and at 320 nm for **1a**. The Peak area was used to quantify each analyte.

### 4.5. In Vivo Anti-Inflammatory Testing

#### 4.5.1. The TPA-Induced Rat Ear Inflammation Model

TPA-induced mouse ear edema was obtained using male Wistar CD-1 mice purchased from Circulo ADN S.A. de C.V., Coyoacan D.F., Mexico (*n* = 3 for each of the flavanones **1a**–**1d**, 20 to 25 g) following the protocol previously described [61]. Edema was induced by the topical application of 2.5 µg of TPA (12-*O*-tetradecanoylphorbol-13-acetate, Sigma, Germany) dissolved in 20 µL ethanol per ear (10 µL each ear side). The standard drug indomethacin was used as a reference. It was dissolved in acetone and put simultaneously on both sides of the right ear (1 mg/ear) with TPA. In the same way, 1 mg of each flavanone (**1a**–**1d**) was dissolved in acetone and then applied on both sides of the right ear with TPA at once, while at the same time, acetone was applied on both sides of the left ear. Four hours after the flavanone solutions had been applied, the animals were sacrificed by dislocation of their necks. Subsequently, the left and right ears were perforated by punching bear (7 mm diameter), and the resulting tissues were accurately weighed. The percent of inhibition of edema formation was assessed according to the following Equation (1):

(1) $$Inhibition\;\left(\%\right)=\frac{difference\;in\;weight\;of\;ear,\;control-difference\;in\;weight\;of\;ear,\;treated}{difference\;in\;weight\;of\;ear,\;control}\times 100$$

The studies were conducted under a protocol in accordance with the Mexican Official Norm for Animal Care and Handling (NOM-062-ZOO-1999) and with the approval of the Academic Committee of Ethics of the Vivarium of the Autonomous University of Morelos State, Mexico, with number 0122013.

#### 4.5.2. Arachidonic Acid (AA)-Induced Rat Ear Inflammation Model

The anti-inflammatory effects of the FS**1**, FS**1a**–FS**1d** were assessed using the AA-induced rat ear edema model as performed in previous studies [34,62] by analyzing histological scores and the cytokine expression by RT-qPCR. In summary, the study protocol using adult male Sprague Dawley^®^ rats was approved by the Ethics Committee of Animal Experimentation of the University of Barcelona (the guidelines for the experiments followed are stated in the protocol “Principles of Laboratory Animal Care” publication 214/97 of 30 July). At first, 5 mg of AA were dissolved in 1 mL of phosphate buffered saline solution. Then, 60 µL of AA solution was applied on both sides of all the ears of the animals (*n* = 5 for each treatment, 200–240 g), except the negative control group (Control -), to induce the inflammatory process with 20 min of exposure. The animals in the positive control (Control +) were treated only with AA solution. Moreover, the other groups were treated with 50 µL of the respective flavanone solution (FS**1**, FS**1a**, FS**1b**, FS**1c**, and FS**1d**) for 20 min. A solution of diclofenac sodium (5 mg/mL, 50 µL) in EtOH/H_2_O (7:3) was used as a reference drug solution (RS). The ear thickness was verified with a digital micrometer (Wisamic Digital Thickness Gauge 0–12.7 mm) in basal state, then measured again after inducing inflammation with AA and, thirdly, after the different treatments with the FS. The edema reduction was calculated by the following Equation (2) [25]:

(2) $$∆\;Inhibition\;inflammation=\frac{difference\;in\;thickness,\;positive\;control-difference\;in\;thickness\;after\;treatment}{difference\;in\;thickness,\;positive\;control}$$

In the same way, the stratum corneum hydration (SCH, arbitrary units AU) of the rat ears was calculated by the difference between SCH value in basal state and the SCH value measured after the AA application and treatments. The measurement was performed with a corneometer CM825 (Courage & Khazaka electronics GmbH, Köln, Germany).

In addition, the left ears of the rats treated with FS**1**, FS**1a**–FS**1d** and the corresponding controls were cut off, rinsed with PBS pH 7.4 and set for 24 h in 4% buffered formaldehyde. Finally, the tissues were dehydrated and embedded in paraffin wax and the ear inflammation was then analyzed under microscope (BX41 microscope and XC50 camera, Olympus Hamburg, Germany) in 5 µm transversal sections stained with hematoxylin and eosin on blind-coded samples.

#### 4.5.3. Gene Expression Analysis by RT-qPCR

Sections of right ear rat tissue from each animal group were homogenized in 1 mL of ice-cold TRI Reagent^®^ (Sigma Aldrich, Madrid, Spain) for 3 min using the Polytron^TM^ Homogenizer PT1200E (Thermo Fisher Scientific, Waltham, MA, USA). Total RNA was isolated using the TRIZol method (Thermo Fisher Scientific, Waltham, MA, USA) following the manufacturer’s protocol. Purity and RNA concentration were measured with a Thermo Scientific Nano Drop TM 2000/2000c Spectrophotometer (Thermo Fisher Scientific, Waltham, MA, USA).

Total RNA (1 µg) was reverse transcribed to cDNA using a High Capacity cDNA Reverse Transcription kit (Applied Biosystems) in a final volume of 20 µL following the manufacturer’s recommendations. qPCR was performed using the StepOne Plus PCR cycler (Applied Biosystems) by using a SYBR^®^ Green PCR Master Mix (Applied Biosystems) and specific oligonucleotides for interleukin-6 (IL-6), interleukin-1β (IL-1β), tumor necrosis factor-α (TNF-α), and the housekeeping β-actin (Table 3). The standard PCR program used was as follows: one denaturation cycle for 10 min at 95 °C followed by 40 cycles of 15 s at 95 °C and 1 min at 60 °C. Relative gene expression of each gene was normalized to β-actin, and the 2^−ΔΔCt^ formula was used to calculate fold-change.

#### 4.5.4. Statistical Analysis

Results are presented as the mean ± standard deviation (SD). Statistical analysis regarding the cytokine study was assessed by one-way analysis of variance (ANOVA), followed by the Tukey’s multiple-comparison post hoc test. The accepted level of significance for the test was *p* < 0.05 value. GraphPad Prism^®^ software (version 5, GraphPad Software, San Diego, CA, USA) was used for all statistical calculations.

## 5. Conclusions

This study shows the anti-inflammatory potential of the hydro alcoholic solutions of natural flavanone **1** and its flavanones **1a**, **1b**, **1c**, and **1d** (FS**1,** FS**1a**–FS**1d**) in the skin. We found a good correlation between the in silico study and the in vivo results. In animal models of acute inflammation, these flavanones ameliorate edema and trigger downregulation of the pro-inflammatory cytokines TNF-α, IL-1β, and IL-6. The results allowed us to recognize the importance of molecular structure in deriving an anti-inflammatory action on skin.

## Figures and Tables

**Table 1:** Predicted activity anti-inflammatory spectrum and Molinspiration^®^ bioactivity score data for chemical structure of flavanones **1**, **1a**–**d**.

Data	Flavanone					Diclofenac	Indomethacin
	1	1a	1b	1c	1d		
Anti-inflammatory (*Pa*)	0.66	0.72	0.61	0.74	0.62	0.79	0.71
miLog*P*	4.49	3.82	4.56	4.22	4.07	4.57	3.99
TPSA	66.76	78.92	55.77	55.77	55.77	49.33	68.54
natoms	23	29	24	24	24	19	25
MW	310.35	394.42	324.38	324.38	322.36	296.15	357.79
Volume	282.78	355.81	300.31	295.6	289.41	238.73	303.24

*Pa*: probability to be active; *P*: octanol–water partition coefficient, TPSA: topological polar surface area; natoms: number of atoms; MW: molecular weight.

**Table 2:** In vivo anti-inflammatory efficacy after TPA (12-*O*-tetradecanoylphorbol 13-acetate) induced mouse edema. Mean ± SD (*n* = 3).

Solutions	FS1	FS1a	FS1b	FS1c	FS1d	Indomethacin
% Inhibition	66.67 ± 1.55	10.27 ± 0.21	25.69 ± 0.52	40.61 ± 0.81	96.27 ± 1.93	91.35 ± 0.47

**Table 3:** Primer sequences used for quantitative RT-qPCR in Sprague Dawley^®^ rats.

Gene (Rat)	Primer Sequences (5′-3′)	Gene Accession Number
IL-6	FW: AGAAAAGAGTTGTGCAATGGCA RV: GGCAAATTTCCTGGTTATATCC	NM_012589.2
TNF-α	FW: AAATGGGCTCCCTCTCATCAGTTC RV: TCTGCTTGGTGGTTTGCTACGAC	NM_012675.3
IL-1β	FW: CACCTCTCAAGCAGAGCACAG RV: GGGTTCCATGGTGAAGTCAAC	NM_031512.2
β-actin	FW: AAGTCCCTCACCCTCCCAAAA RV: AAGCAATGCTGTCACCTTCCC	V01217.1

## References

[1] Abdel-Mottaleb M.M., Try C., Pellequer Y., Lamprecht A. (2014). Nanomedicine strategies for targeting skin inflammation. Nanomedicine.

[2] Maleki S.J., Crespo J.F., Cabanillas B. (2019). Anti-inflammatory effects of flavonoids. Food Chem..

[3] Paoletti T., Fallarini S., Gugliesi F., Minassi A., Appendino G.B., Lombardi G. (2009). Anti-inflammatory and vascular protective properties of 8-prenylapigenin. Eur. J. Pharmacol..

[4] Park K.E., Qin Y., Bavry A.A. (2012). Nonsteroidal anti-inflammatory drugs and their effects in the elderly. Aging Health.

[5] Li Y.-S., Zhang J., Tian G.-H., Shang H.-C., Tang H.-B. (2021). Kirenol, darutoside and hesperidin contribute to the anti-inflammatory and analgesic activities of *Siegesbeckia pubescens* makino by inhibiting COX-2 expression and inflammatory cell infiltration. J. Ethnopharmacol..

[6] Keseroglu H.O., Gonul M. (2014). Traditional topical herbal therapies in psoriasis. Tang Humanit. Med..

[7] Dhingra A.K., Chopra B., Bonthagarala B. (2018). Natural Anti-Inflammatory Agents: Recent Progress and Future Perspectives. Ann. Pharmacol. Pharm..

[8] Singh M.R., Nag M.K., Patel S., Daharwal S.J. (2013). Novel Approaches for Dermal and Transdermal Delivery of Herbal Drugs. J. Pharmacogn. Phytochem..

[9] Scheau C., Badarau I.A., Mihai L.-G., Scheau A.-E., Costache D.O., Constantin C., Calina D., Caruntu C., Costache R.S., Caruntu A. (2020). Cannabinoids in the Pathophysiology of Skin Inflammation. Molecules.

[10] Kim H.P., Son K.H., Chang H.W., Kang S.S. (2020). Anti-inflammatory Plant Polyphenolics and Cellular Action Mechanisms. Curr. Bioact. Compd..

[11] Chen J., Li W., Yao H., Xu J. (2015). Insights into drug discovery from natural products through structural modification. Fitoterapia.

[12] Deenonpoe R., Prayong P., Thippamom N., Meephansan J., Na-Bangchang K. (2019). Anti-inflammatory effect of naringin and sericin combination on human peripheral blood mononuclear cells (hPBMCs) from patient with psoriasis. BMC Complement. Altern. Med..

[13] Al-Roujayee A.S. (2017). Naringenin improves the healing process of thermally-induced skin damage in rats. J. Int. Med. Res..

[14] Chi Y.S., Lim H., Park H., Kim H.P. (2003). Effects of wogonin, a plant flavone from *Scutellaria radix*, on skin inflammation: In vivo regulation of inflammation-associated gene expression. Biochem. Pharmacol..

[15] Alalaiwe A., Lin C.-F., Hsiao C.-Y., Chen E.-L., Lin C.-Y., Lien W.-C., Fang J.-Y. (2020). Development of flavanone and its derivatives as topical agents against psoriasis: The prediction of therapeutic efficiency through skin permeation evaluation and cell-based assay. Int. J. Pharm..

[16] Barreca D., Gattuso G., Bellocco E.S., Calderaro A., Trombetta D., Smeriglio A., Laganà G., Daglia M., Meneghini S., Nabavi S.M. (2017). Flavanones: Citrus phytochemical with health-promoting properties. BioFactors.

[17] Garcia-Campoy A., Garcia E., Muñiz-Ramirez A. (2020). Phytochemical and Pharmacological Study of the *Eysenhardtia* Genus. Plants.

[18] Narváez-Mastache J.M., Soto C., Delgado G. (2007). Antioxidant Evaluation of *Eysenhardtia* Species (Fabaceae): Relay Synthesis of 3-*O*-Acetyl-11α, 12α-epoxy-oleanan-28, 13β-olide Isolated from *E. platycarpa* and Its Protective Effect in Experimental Diabetes. Biol. Pharm. Bull..

[19] Wächter G.A., Hoffmann J.J., Furbacher T., Blake M.E., Timmermann B.N. (1999). Antibacterial and antifungal flavanones from *Eysenhardtia texana*. Phytochemistry.

[20] Pérez-Guitierrez R.M., Vargas-solís R., García-Dueñas L.M., Dávila-Badillo L. (2002). Efecto de isoflavonas aisladas de la corteza de *Eysenhardtia polystachya* sobre el crecimiento de cristales de oxalato y fosfato de calcio urinario. Boletín del Col. Mex. Urol..

[21] Alvarez L., Rios M.Y., Esquivel C., Chávez M.I., Delgado G., Aguilar M.I., Villarreal M.L., Navarro V. (1998). Cytotoxic Isoflavans from *Eysenhardtia polystachya*. J. Nat. Prod..

[22] Domínguez-Villegas V., Domínguez-Villegas V., García M.L., Calpena A.C., Clares-Naveros B., Garduño-Ramirez M.L. (2013). Anti-inflammatory, Antioxidant and Cytotoxicity Activities of Methanolic Extract and Prenylated Flavanones Isolated from Leaves of *Eysehardtia platycarpa*. Nat. Prod. Commun..

[23] Shi L., Feng X.E., Cui J.R., Fang L.H., Du G.H., Li Q.S. (2010). Synthesis and biological activity of flavanone derivatives. Bioorg. Med. Chem. Lett..

[24] Gordaliza M. (2007). Natural products as leads to anticancer drugs. Clin. Transl. Oncol..

[25] Li S., Xiong Q., Lai X., Li X., Wan M., Zhang J., Yan Y., Cao M., Lu L., Guan J. (2015). Molecular Modification of Polysaccharides and Resulting Bioactivities. Compr. Rev. Food Sci. Food Saf..

[26] Filimonov D., Lagunin A.A., Gloriozova T.A., Rudik A., Druzhilovskii D.S., Pogodin P.V., Poroikov V.V. (2014). Prediction of the Biological Activity Spectra of Organic Compounds Using the Pass Online Web Resource. Chem. Heterocycl. Compd..

[27] Nadeem S., Sirajuddin M., Ahmad S., Tirmizi S.A., Ali M.I., Hameed A. (2016). Synthesis, spectral characterization and in vitro antibacterial evaluation and Petra/Osiris/Molinspiration analyses of new Palladium(II) iodide complexes with thioamides. Alex. J. Med..

[28] Farias I.V., Faqueti L.G., Noldin V.F., Junior G.F., Nowil A.E., Schuquel I.T., Monache F.D., García P.A., López-Pérez J.L., Feliciano A.S. (2018). Cytotoxic phloroglucinol meroterpenoid from *Eugenia umbelliflora* fruits. Phytochem. Lett..

[29] Jarrahpour A., Fathi J., Mimouni M., Hadda T.B., Sheikh J., Chohan Z., Parvez A. (2011). Petra, Osiris and Molinspiration (POM) together as a successful support in drug design: Antibacterial activity and biopharmaceutical characterization of some azo Schiff bases. Med. Chem. Res..

[30] Baskar V., Jayalakshmi C., Pavithra N., Veronica Grite S. (2014). Validating therapeutically active phytochemical compounds for anti-ageing by in silico pharmacokinetic approach. J. Biol. Inf. Sci..

[31] Yi Y.-S. (2021). Flavonoids: Nutraceuticals for Rheumatic Diseases via Targeting of Inflammasome Activation. Int. J. Mol. Sci..

[32] Chibli L.A., Rodrigues K.C., Gasparetto C.M., Pinto N.C., Fabri R.L., Scio E., Alves M.S., Del-Vechio-Vieira G., Sousa O.V. (2014). Anti-inflammatory effects of *Bryophyllum pinnatum* (Lam.) Oken ethanol extract in acute and chronic cutaneous inflammation. J. Ethnopharmacol..

[33] Siddiqui F., Naqvi S., Abidi L., Faizi S., Avesi L., Mirza T., Farooq A.D. (2016). Opuntia dillenii cladode: Opuntiol and opuntioside attenuated cytokines and eicosanoids mediated inflammation. J. Ethnopharmacol..

[34] Bustos-Salgado P., Andrade-Carrera B., Domínguez-Villegas V., Rodríguez-Lagunas M.J., Boix-Montañes A., Calpena-Campmany A., Garduño-Ramírez M.L. (2021). Biopharmaceutic study and in vivo efficacy of natural and derivatives flavanones formulations. Nanomedicine.

[35] Gomes A., Fernandes E., Lima J., Mira L., Corvo M.L. (2008). Molecular Mechanisms of Anti-Inflammatory Activity Mediated by Flavonoids. Curr. Med. Chem..

[36] Gautam R., Jachak S.M. (2009). Recent developments in anti-inflammatory natural products. Med. Res. Rev..

[37] Otuki M.F., Vieira-Lima F., Malheiros Â., Yunes R.A., Calixto J.B. (2005). Topical anti-inflammatory effects of the ether extract from Protium kleinii and α-amyrin pentacyclic triterpene. Eur. J. Pharmacol..

[38] Ren X., Zhang M., Chen L., Zhang W., Huang Y., Luo H., Li L., He H. (2017). The anti-inflammatory effects of Yunnan Baiyao are involved in regulation of the phospholipase A2/arachidonic acid metabolites pathways in acute inflammation rat model. Mol. Med. Rep..

[39] Sanaki T., Kasai-Yamamoto E., Yoshioka T., Sakai S., Yuyama K., Fujiwara T., Numata Y., Igarashi Y. (2017). Direct Involvement of Arachidonic Acid in the Development of Ear Edema via TRPV3. J. Oleo Sci..

[40] Rodrigues K.C.M., Chibli L.A., Santos B.C.S., Temponi V.S., Pinto N.C.C., Scio E., Del-Vechio-Vieira G., Alves M.S., Sousa O.V. (2016). Evidence of Bioactive Compounds from *Vernonia polyanthes* Leaves with Topical Anti-Inflammatory Potential. Int. J. Mol. Sci..

[41] Griswold D.E., Martin L.D., Badger A.M., Breton J., Chabot-Fletcher M. (1998). Evaluation of the cutaneous anti-inflammatory activity of azaspiranes. Inflamm. Res..

[42] Passos G.F., Medeiros R., Marcon R., Nascimento A.F., Calixto J.B., Pianowski L.F. (2013). The role of PKC/ERK1/2 signaling in the anti-inflammatory effect of tetracyclic triterpene euphol on TPA-induced skin inflammation in mice. Eur. J. Pharmacol..

[43] (2002). Gábor, MThe mouse ear as a model for cutaneous irritation. J. Toxicol. Cutan. Ocul. Toxicol..

[44] Saraiva R.A., Araruna M.K., Oliveira R.C., Menezes K.D., Leite G.O., Kerntopf M.R., Costa J.G., Rocha J.B., Tomé A.R., Campos A.R. (2011). Topical anti-inflammatory effect of *Caryocar coriaceum* Wittm. (Caryocaraceae) fruit pulp fixed oil on mice ear edema induced by different irritant agents. J. Ethnopharmacol..

[45] Peinnequin A., Mouret C., Birot O., Alonso A., Mathieu J., Clarençon D., Agay D., Chancerelle Y., Multon E. (2004). Rat pro-inflammatory cytokine and cytokine related mRNA quantification by real-time polymerase chain reaction using SYBR green. BMC Immunol..

[46] Spies M., Nesic O., Barrow R.E., Perez-Polo J.R., Herndon D.N. (2001). Liposomal IGF-1 gene transfer modulates pro- and anti-inflammatory cytokine mRNA expression in the burn wound. Gene Ther..

[47] Xiao S., Yu H., Xie Y., Guo Y., Fan J., Yao W. (2021). The anti-inflammatory potential of *Cinnamomum camphora* (L.) J.Presl essential oil in vitro and in vivo. J. Ethnopharmacol..

[48] Giongo J.L., Vaucher R.D.A., Sagrillo M.R., Santos R.C.V., Duarte M.M., Rech V.C., Lopes L.Q.S., da Cruz I., Tatsch E., Moresco R.N. (2017). Anti-inflammatory effect of geranium nanoemulsion macrophages induced with soluble protein of *Candida albicans*. Microb. Pathog..

[49] Wang H., Peters T., Kess D., Sindrilaru A., Oreshkova T., Van Rooijen N., Stratis A., Renkl A.C., Sunderkötter C., Wlaschek M. (2006). Activated macrophages are essential in a murine model for T cell-mediated chronic psoriasiform skin inflammation. J. Clin. Investig..

[50] Yao F., Xue Q., Li K., Cao X., Sun L., Liu Y. (2019). Phenolic Compounds and Ginsenosides in Ginseng Shoots and Their Antioxidant and Anti-Inflammatory Capacities in LPS-Induced RAW264.7 Mouse Macrophages. Int. J. Mol. Sci..

[51] Owona B.A., Abia W.A., Moundipa P.F. (2020). Natural compounds flavonoids as modulators of inflammasomes in chronic diseases. Int. Immunopharmacol..

[52] Denaro M., Smeriglio A., Trombetta D. (2021). Antioxidant and anti-inflammatory activity of citrus flavanones mix and its stability after in vitro simulated digestion. Antioxidants.

[53] Tejada S., Pinya S., Martorell M., Capó X., Tur J.A., Pons A., Sureda A. (2019). Potential Anti-inflammatory Effects of Hesperidin from the Genus Citrus. Curr. Med. Chem..

[54] Pahl H.L. (1999). Activators and target genes of Rel/NF-kappaB transcription factors. Oncogene.

[55] Sarango-Granda P., Silva-Abreu M., Calpena A., Halbaut L., Fábrega M.-J., Rodríguez-Lagunas M., Díaz-Garrido N., Badia J., Espinoza L. (2020). Apremilast Microemulsion as Topical Therapy for Local Inflammation: Design, Characterization and Efficacy Evaluation. Pharmaceuticals.

[56] Xian Y.-F., Hu Z., Ip S.-P., Chen J.-N., Su Z.-R., Lai X.-P., Lin Z.-X. (2018). Comparison of the anti-inflammatory effects of *Sinapis alba* and *Brassica juncea* in mouse models of inflammation. Phytomedicine.

[57] Andrade-Carrera B., Clares B., Noé V., Mallandrich M., Calpena A.C., García M.L., Garduño-Ramírez M.L. (2017). Cytotoxic Evaluation of (2S)-5,7-Dihydroxy-6-prenylflavanone Derivatives Loaded PLGA Nanoparticles against MiaPaCa-2 Cells. Molecules.

[58] Tariq M., Sirajuddin M., Ali S., Khalid N., Tahir M.N., Khan H., Ansari T.M. (2016). Pharmacological investigations and Petra/Osiris/Molinspiration (POM) analyses of newly synthesized potentially bioactive organotin(IV) carboxylates. J. Photochem. Photobiol. B Biol..

[59] Imran M., Aziz M., Kumar N., Kousar Z., Shabnam S., Nohri F. (2016). Synthesis, spectroscopic characterization and Petra Osiris Molinspiration (POM) analyses of dicarboxylic acid amides. Int. J. Pharm. Sci. Res..

[60] Bustos-Salgado P., Andrade-Carrera B., Garduño-Ramírez M.L., Alvarado H., Calpena-Campmany A. (2020). Quantification of one Prenylated Flavanone from *Eysenhardtia platycarpa* and four derivatives in Ex Vivo Human Skin Permeation Samples Applying a Validated HPLC Method. Biomolecules.

[61] Rincón M., Calpena A.C., Clares B., Espina M., Garduño-Ramírez M.L., Rodríguez-Lagunas M.J., García M.L., Abrego G. (2018). Skin-controlled release lipid nanosystems of pranoprofen for the treatment of local inflammation and pain. Nanomedicine.

[62] Espinoza L.C., Silva-Abreu M., Calpena A.C., Rodríguez-Lagunas M.J., Fábrega M.-J., Garduño-Ramírez M.L., Clares B. (2019). Nanoemulsion strategy of pioglitazone for the treatment of skin inflammatory diseases. Nanomed. Nanotechnol. Biol. Med..

